# Malaria According to the New Researches

**Published:** 1901-03

**Authors:** 


**Malaria according to the New Researches.** By Professor Angelo Celli. Pp. xxiv., 275. London: Longmans, Green, & Co. 1900.

This monograph, translated from the second Italian edition by John Joseph Eyre, D.P.H. Cambridge, "informs us fully of what the Italians have done and of what they are doing in the study, and in the prophylaxis, of malaria." Professor Celli's work appeals to English-speaking peoples even more than to his own countrymen, inasmuch as the interests of the latter in this respect are insignificant as compared with those of Great Britain and the United States. It is not quite sufficiently realised in this country that, whatever the surrounding malaria-haunted Campagna may be, "Rome is now not only one of the healthiest cities in the world, but also one of the most desirable health resorts in Europe for certain forms of disease."

In an interesting introduction to the volume, Dr. Patrick Manson points out the fact that "we owe to the Italians not only the name but also much of our knowledge of Malaria," and that "when, two years ago, Ross's brilliant investigations placed the mosquito-malaria theory on an incontrovertible basis, they were the first to confirm his work, to extend it, and to throw themselves energetically into the hygienic measures it so plainly indicated."

The whole basis of the volume rests upon the now abundantly proved dictum that malaria must no longer be considered a miasmatic disease, but a typically contagious one; that is, a contagion which is transmitted from man to man, not directly, but by the agency of the mosquito. It will be interesting to many victims to know that the most efficient culicide is tobacco-smoke.

